# SEOM clinical guideline of diagnosis and management of low-grade glioma (2017)

**DOI:** 10.1007/s12094-017-1790-3

**Published:** 2017-11-09

**Authors:** J. M. Sepúlveda-Sánchez, J. Muñoz Langa, M. Á. Arráez, J. Fuster, A. Hernández Laín, G. Reynés, V. Rodríguez González, E. Vicente, M. Vidal Denis, Ó. Gallego

**Affiliations:** 1 Neurooncology Unit, Hospital Universitario, 12 de Octubre, Madrid, Spain; 2 Medical Oncology Department, Hospital Universitari I Politècnic la Fe, Valencia, Spain; 3 Neurosurgery Department, HRU Carlos Haya, Málaga, Spain; 4 Medical Oncology Department, Hospital Universitari Son Espases, Palma de Mallorca, Spain; 5 Neuropathology Department, Hospital Universitario, 12 de Octubre, Madrid, Spain; 6 Radiation Oncology Department, Hospital Universitario, 12 de Octubre, Madrid, Spain; 7 Medical Oncology Department, C.H.U. Insular-Materno Infantil de Gran Canaria, Las Palmas, Spain; 8 Neuroradiology Unit, HRU Carlos Haya, Málaga, Spain; 9 Medical Oncology Department, Hospital de la Santa Creu I Sant Pau, Barcelona, Spain

**Keywords:** Astrocytoma, Oligodendroglioma, Low-grade glioma, Guideline, Neurooncology

## Abstract

Diffuse infiltrating low-grade gliomas include oligodendrogliomas and astrocytomas, and account for about 5% of all primary brain tumors. Treatment strategies for these low-grade gliomas in adults have recently changed. The 2016 World Health Organization (WHO) classification has updated the definition of these tumors to include their molecular characterization, including the presence of isocitrate dehydrogenase (IDH) mutation and 1p/19p codeletion. In this new classification, the histologic subtype of grade II-mixed oligoastrocytoma has also been eliminated. The precise optimal management of patients with low-grade glioma after resection remains to be determined. The risk–benefit ratio of adjuvant treatment must be weighed for each individual.

## Introduction

Diffuse infiltrating low-grade gliomas include oligodendrogliomas and astrocytomas and account for about 5% of all primary brain tumors. Treatment strategies for these low-grade gliomas (LGG) in adults have recently changed. The 2016 World Health Organization (WHO) classification has updated the definition of these tumors to include their molecular characterization, including the presence of isocitrate dehydrogenase (IDH) mutation and 1p/19p codeletion. In this new classification, the histologic subtype of grade II-mixed oligoastrocytoma has been eliminated.

Current recommendations for complementary treatment following surgery are based on moderate and controversial evidence. Most of the clinical studies that have attempted to address the role and timing of radiotherapy and chemotherapy have included different mixtures of patients with astrocytic and oligodendroglial tumors that complicate their interpretation. In addition, the recent molecular markers, IDH and 1p/19q codeletion, were not taken into account when designing the historical clinical trials.

LGG are associated with a wide range of symptoms, such as seizures, cognitive impairment, paresis or brain edema, with a challenging management that requires a multidisciplinary team including neurosurgeons, medical and radiotherapy oncologists, neurologists, neuroradiologists and neuropathologist.

## Methodology

These guidelines have been developed by a multidisciplinary group with expertise in clinical and investigational neurooncology. A bibliographic search of published articles was performed in the MEDLINE database (PubMed). The different sections were written by different responsible experts and afterwards all the authors discussed the results and determined the level of evidence described in Table 1 [1].

**Table 1:** Levels of evidence/grades of recommendation

Levels of evidence
I. Evidence from at least one large randomized, controlled trial of good methodological quality (low potential for bias) or meta-analyses of well-conducted randomized trials without heterogeneity
II. Small randomized trials or large randomized trials with a suspicion of bias (lower methodological quality) or meta-analyses of such trials or of trials with demonstrated heterogeneity
III. Prospective cohort studies
IV. Retrospective cohort studies or case–control studies
V. Studies without control group, case reports, experts opinions
Grades of recommendation
A. Strong evidence for efficacy with a substantial clinical benefit, strongly recommended
B. Strong or moderate evidence for efficacy but with a limited clinical benefit, generally recommended
C. Insufficient evidence for efficacy or benefit does not outweigh the risk or the disadvantages; optional
D. Moderate evidence against efficacy or for adverse outcome, generally not recommended
E. Strong evidence against efficacy or for adverse outcome, never recommended

The aim of this document is to provide a clear practical recommendation for the management of low-grade gliomas in Spain.

## Histological and molecular diagnosis and classification

The histological evaluation of the glioma subtype is based on the morphological similarity of the tumor cells to normal glial cell found in the brain. LGG are separated into astrocytomas and oligodendrogliomas. Those with uniformly rounded nuclei and perinuclear halo (“fried egg”) are considered oligodendrogliomas, while those with nuclear irregularities with fibrillary processes are diagnosed as astrocytomas. A variant of diffuse astrocytic tumor is gemistocytic astrocytoma, characterized by > 20% cells with abundant eccentrically placed cytoplasm, this variant tends to show a rapid malignant progression.

The new WHO classification 2016 discourages the diagnosis of mixed oligoastrocytoma since molecular studies have demonstrated either an astrocytic or an oligodendroglial genotype, implying that these tumors do not constitute a separate entity [2].

For grading of diffuse gliomas, the histologic features of mitotic activity, microvascular proliferation and necrosis are used. The diagnosis of diffuse astrocytoma (WHO grade II) requires the absence of increased mitotic activity, microvascular proliferation or necrosis. A single mitosis in a large resection specimen is compatible with grade II, whereas it may well indicate anaplasia (WHO grade III) in a small sample.

The diagnosis of oligodendroglioma (WHO grade II) requires the absence of pathological microvascular proliferation and brisk mitotic activity (≤ 6/10 HPF).

### Molecular biology

The revised WHO classification of 2016 integrates molecular markers in the routine histological diagnosis of CNS tumors. If molecular testing cannot be performed, the term “not otherwise specified (NOS)” should be added.

The traditional histologic separation between astrocytomas and oligodendrogliomas is complemented by recent evidence for two molecularly and virtually exclusive subtypes, characterized by IDH, ATRX and TP53 mutation in the absence of 1p/19q codeletion (astrocytic) versus IDH mutation, 1p/19q codeletion, and TERT promoter mutation (oligodendroglial).

Diffuse astrocytomas are now divided into IDH-mutant, IDH-wildtype and NOS categories. The great majority of grade II astrocytic tumors falls into the IDH-mutant category (from 59 to 90%). Demonstration of the ATRX mutation or loss of ATRX nuclear expression is recommended for the diagnosis of astrocytoma [3]. IDH-wildtype diffuse astrocytomas most likely constitute a variety of entities that could be reclassified with additional genetic analysis. Thus, IDH-wildtype diffuse astrocytoma is considered a provisional entity.

The diagnosis of oligodendroglioma requires the demonstration of both an IDH gene mutation and combined whole-arm 1p/19q codeletion.

### Diagnosis algorithm

A stepwise diagnosis algorithm is recommended, starting with immunochemistry for ATRX and R132H-mutant IDH1 (representing about 90% of all IDH mutations), followed by testing for 1p/19q codeletion, and then followed by IDH sequencing of the tumors that were negative for IDH1 immunochemistry [3].

**Fig. 1:**
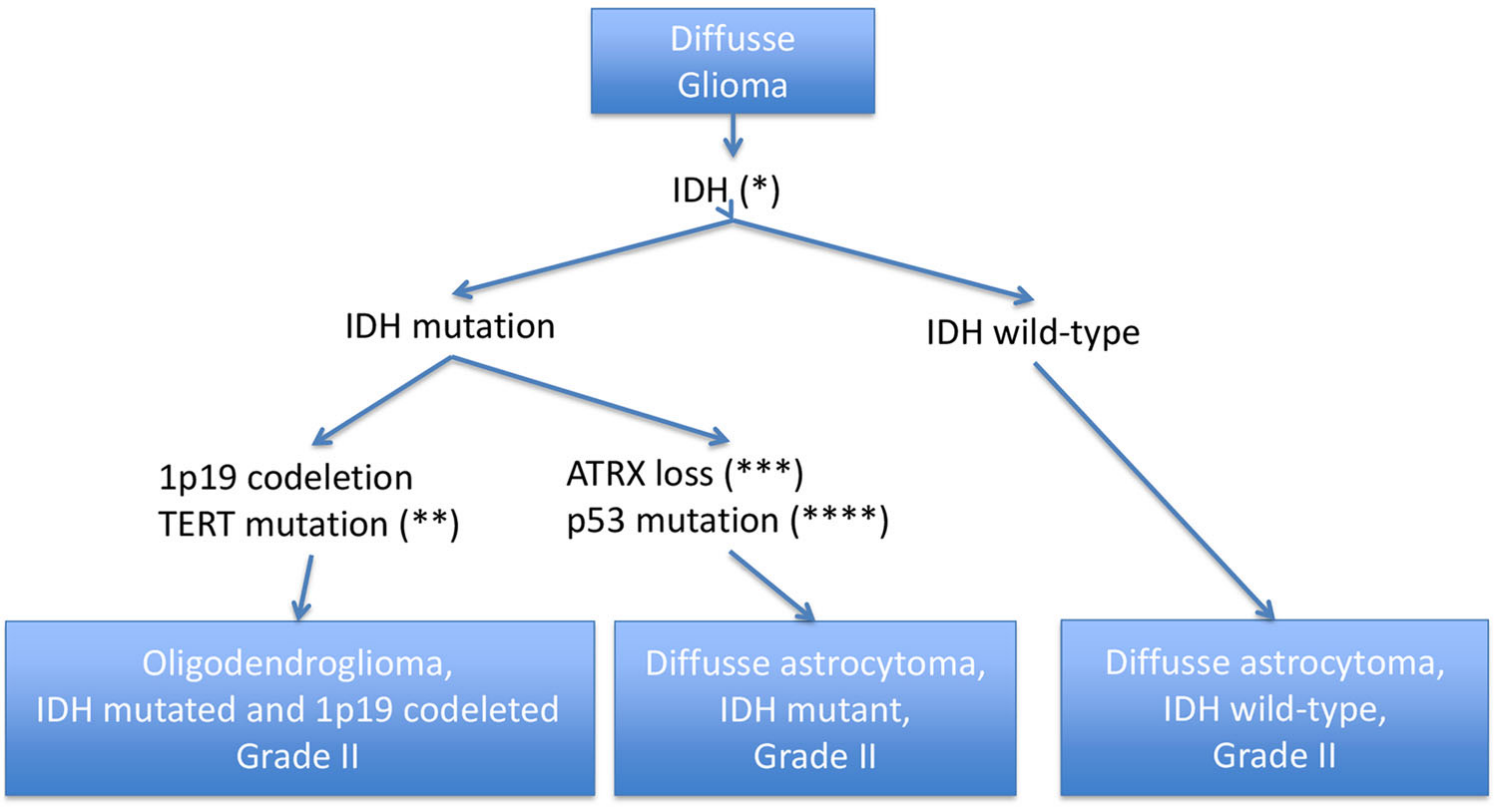
*It is recommended to start with immunochemistry for R132H-mutant IDH1 followed by IDH1 and IDH2 sequencing of the tumors that were negative for R132H-mutant IDH1 by immunochemistry. **Characteristic but not necessary for diagnosis. ***Recommended to confirm the astrocytic subtype but not necessary for diagnosis. Usually performed with immunochemistry. ****Characteristic but not necessary for diagnosis

With this approach, the majority of LGG can be assigned to one of the three mayor classes: IDH-mutant astrocytoma, IDH-mutant and 1p/19q-codeleted oligodendroglioma and IDH-wildtype gliomas which commonly demonstrate similar genetic alterations of glioblastoma and the associated poor prognosis (Fig. 1).

Recent studies have challenged the prognostic value of WHO grading by demonstrating similar overall survival (OS) of patients with IDH-mutant WHO grade II and WHO grade III gliomas [4]. For the time being, the WHO classification of 2016 recommends retaining traditional histologic grading of diffuse gliomas. Future studies need to clarify whether grading should be modified by molecular testing.

## Radiological features and evaluation

On conventional radiological imaging, such as CT and MRI, different subtypes of LGG are very similar, often indistinguishable.

On CT, LGG appear as areas of iso or low attenuation with variable mass effect on adjacent structures, poorly delimited, often without contrast enhancement or perilesional edema. Calcifications are not common (10–20% of cases) and may be related to oligodendroglial components.

MRI is the modality of choice for characterizing these lesions. LGG are often homogeneous with low signal intensity on conventional T1-weighted images and have high signal intensity on T2-weighted sequences. The high T2 signal is not related to cellularity or cellular atypia, but rather oedema, demyelination and other degenerative changes. Cystic components are also encountered. Fluid-Attenuated Inversion Recovery (FLAIR) sequence shows the best contrast between presumed infiltrating tumor margins and normal brain. Commonly, astrocytomas are confined to white matter although they can infiltrate and expand the adjacent cortex in later stages. However, oligodendroglioma is frequently a cortical-based tumor.

Although contrast enhancement has been classically associated with a higher degree of malignancy, contrast enhancement may be seen in up to 20% of LGG. However, grade III and IV gliomas often demonstrate a higher degree of tumor heterogeneity and contrast enhancement than LGG.

### Advanced MRI

Advanced MR imaging techniques, such as Diffusion weighted imaging (DWI), MR Spectroscopy and Perfusion MRI complements, the anatomic information obtained from conventional MRI. Diffusion Tensor Imaging (DTI) and functional MRI also add important information, specifically on surgery planning [5].

DWI quantifies cellularity on the basis of the premise that water diffusivity within the extracellular compartment is inversely related to the content and attenuation of the intracellular space. Therefore, DWI serve as a marker of tumor cellularity. Characteristically, LGGs present low cellularity and non-restricted diffusion.

DTI data and further application using fiber-tracking techniques (tractography) can reveal the microstructural integrity of brain tissue and the relationship between the tumor and adjacent white matter tracts: LGG tend to deviate, rather than destruct or infiltrate the adjacent white matter.

MRI Spectroscopy can, noninvasively, measure the brain metabolites in vivo. Although no tumor specific metabolite has been labeled, the ratios of metabolites have been used to assess cellular proliferation and the mitotic activity: generally, LGG present decreased N-Acetyl-Aspartate (NAA) peak, medium choline peaks, absence of lactate peak and increased myo-inositol [6].

**Fig. 2:**
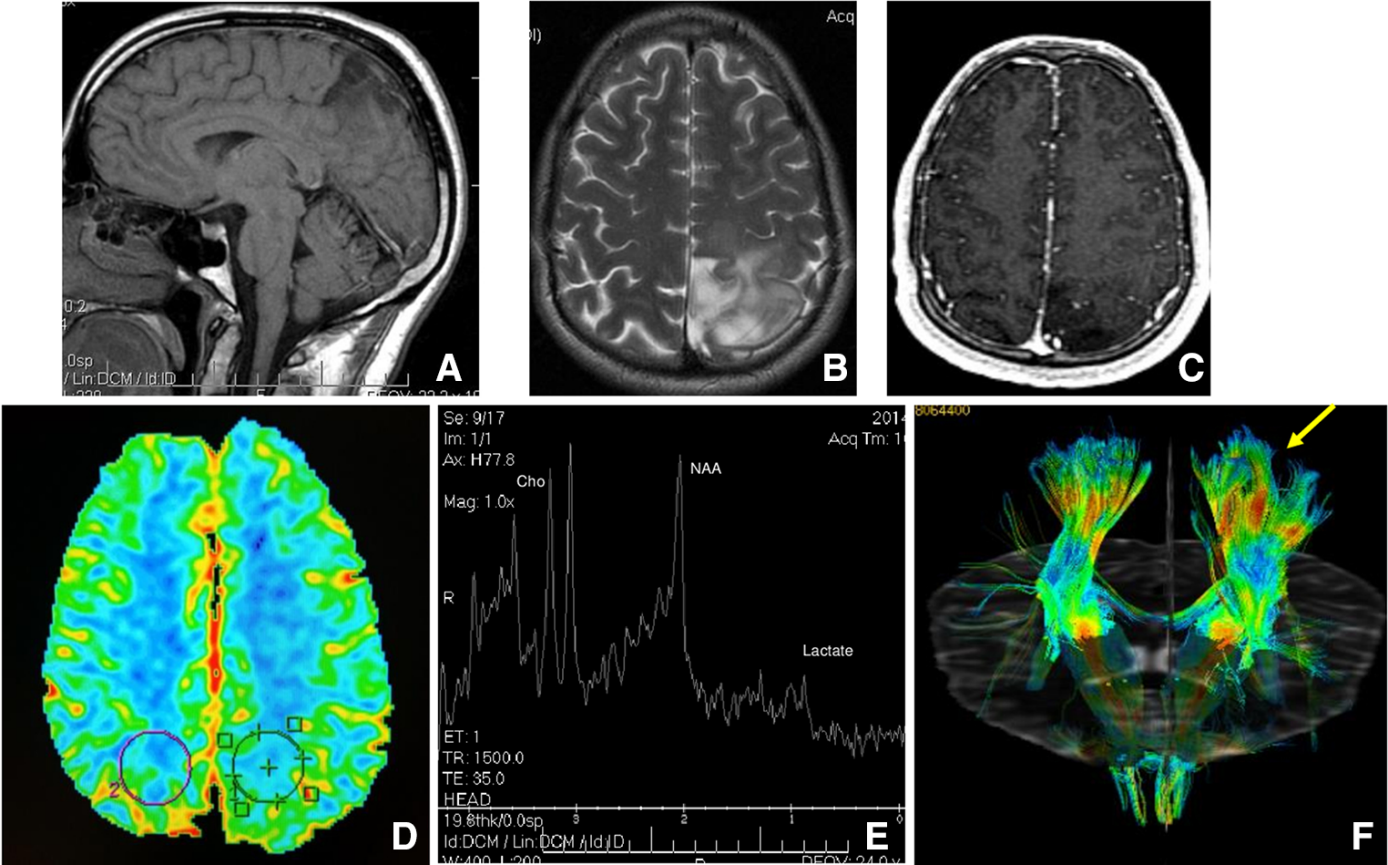
Diffuse grade 2 astrocytoma: Left parietal mass with low T1 signal and high and heterogenious T2 signal with cystic areas withoyt contrast enhancement. Sagital T1 sequence (**a**), Axial T2 (**b**) and T1 postcontrast sequence (**c**). Perfussion MRI shows no elevation of rCBV (**d**). MRI spectroscopy: low NAA peak without lactate peak (**e**). Post process DTI (tractography): Left piramidal tracts displacement (**f**)

Perfusion-weighted MRI generates a series of parameters, including relative cerebral blood volume (rCBV), referring to volume of blood in a given region of brain tissue. rCBV provides a reliable estimation of tumor microvascular density and tumor aggressiveness. LGGs usually show no increase in tumor rCBV: LGG have rCBV values of range between 1.11 and 2.14 (Fig. 2).

### Radiological assessment of response in low grade glioma

Currently, the Response Assessment in Neuro-Oncology (RANO) defines a set of criteria for assessing outcome in trials of diffuse LGG. RANO includes specific guidelines for using tumor size and appearance on T2/FLAIR and T1 weighted MRI sequences on conventional MRI, to define complete response, partial response, stable disease and progression [7].

But the optimal method of assessing treatment response in LGG remains an active area of investigation. The consensus guidelines await validation of RANO criteria in future randomized studies. Some authors suggest that treatment outcomes might be more reliably evaluated using advanced imaging techniques. However, none of these alternative imaging markers have been validated for use in LGG clinical trials or in clinical practice.

In our daily practice, conventional MRI are mandatory adding advanced sequences in difficult cases.

## Surgery in LGG

### Open resection and biopsy

Surgical resection is considered the first step to be done when dealing with LGG. Currently, it is assumed that surgery should aim for the greater extent of resection [8, 9] as it would:

- increase survival and potentially alter the natural history of the disease: gross total removal or subtotal tumor removal (when feasible and safe) is superior to biopsy in terms of decreasing the rate of tumor progression (Level II) and also have positive impact in overall survival (Level III). Preoperative estimation of resection less than 50% would lead to consider biopsy. A retrospective multicentric study analyzing 1097 patients (in which the assessed population was divided into three subgroups depending on the extent of resection: 100%, 50–99% and less than 50%) showed that the amount of residual lesion impacted on the course of the disease (OS was 10.5 and 14 years for patients with a less than 50 and 50–99% Extent of resection, being unreached instead after 15 years for patients with no residual tumor).
- Assure histological diagnosis and molecular analysis (Level III).
- Ameliorated mass effect and intracranial hypertension.
- Ease control of seizures (Level III). Patients with LGG and seizures would have Engel class 1 outcome (seizure-free) in 67–70% of cases and improvement in another 20–25%.

Biopsy is indicated when diagnosis is needed in deep lesions (including brainstem), diffuse and/or multicentric tumor or any other contraindication for open resection. Biopsy can be stereotactic (framed or frameless) or open. Neuronavigated non-framed biopsy is gaining acceptance.

### The issue of incidental LGG

Surgical resection is superior when compared with observation to improve overall survival for patients with LGG (Level III) although observation has no negative impact on cognitive performance and quality of life (Level II) [10].

### Ancillary techniques in surgery of LGG

Neuronavigation, ultrasonography and intraoperative MRI are used to increase resection and diminish morbidity. Preoperative MRI with tensor diffuse images improves postoperative outcome (Level III) in lesions near motor and sensitive tracts. Neurophysiological evaluation, intraoperative mapping and awake surgery are desirable in speech and other eloquent areas to diminish morbidity after surgery (Level III) [10].

### Limits of the evaluation of the surgical treatment

Many studies are retrospective or case-series investigations (level of evidence III–IV). No randomization was made regarding the extent of the surgical resection in many of them. Outcome measures also differed among studies. In addition, the minority of these analyses reported a volumetric assessment of the extent of resection.

Aggressive surgical resection of LGG may improve clinical picture, PFS and OS. Clinical setting evaluation would lead toward open gross resection or biopsy. Preoperative imaging and intraoperative techniques improve resection and diminish morbidity. In spite of these assumptions, levels of evidence are still weak in surgery of LGG.

## Timing of complementary treatment

Surgery alone is not curative in patients with low-grade gliomas, and additional therapy (radiation and/or chemotherapy) is ultimately required in all patients. However, the optimal timing of additional therapy is uncertain and the decision to proceed with immediate versus delayed postoperative therapy must be individualized. Table 2 summarizes the main clinical trials assessing the effectiveness of different adjuvant treatments in LGG.

**Table 2:** Summaries studies regarding adjuvant therapy in Low-grade gliomas (LGG)

Study	Design	Inclusion criteria	Treatment protocol	Results and comments
Observation (wait and see)				
RTOG 9802 (Shaw EG. J Neurosurg 2008) [13]	Phase II (*n* = 111)	Age < 40 years Gross Tumor resection	No adjuvant therapy	OS rates at 2 and 5 years were: 99 and 93%, respectively. PFS rate at 5 year: 48% In patients without unfavorable prognostic factors^a^, 2- and 5-year PFS rates were 100 and 70%., respectively.
Adjuvant radiotherapy				
EORTC 22845/MRC BR04 (Karim AB. Int J Radiat Oncol Biol Phys 2002) [17]	Phase III (*n* = 290)	Low-grade astrocytoma, oligodendroglioma, and mixed oligoastrocytomas	Early RT (54 Gy) versus No postoperative RT	Early RT showed an improvement in TTP (4.8 versus 3.4 years; *p* = 0.02). HR = 0.68 (95% CI 0.50–0.94). No differences in OS: HR = 1.15 (95% CI 0.67–1.74). The 5-year OS rate were: 63 versus 66% (*p* = 0.49).
EORTC 22845 (Van den Bent MJ. Lancet 2005) [16]	Phase III (*n* = 311)	WHO grade II or pilocytic astrocytomas with incomplete resection	RT (54 Gy) after biopsy or initial resection, versus No RT until progression	Early RT was associated with an improvement PFS (5.3 versus 3.4 years): HR = 0.59; 95% CI 0.45–0.77 (*p* = 0.0001). No difference in OS (7.4 versus 7.2 years): HR = 0.97; 95% CI 0.71–1.34 8 (*p* = 0.872) Seizure control also improved in patients treated with early radiotherapy.
EORTC 22844 (Karim AB. Int J Radiat Oncol Biol Phys 1996) [19]	Phase III (*n* = 379)	Low-grade astrocytomas (Gl and G2), oligodendrogliomas, and mixed oligoastrocytomas	Surgery + low dose RT (45 Gy) versus Surgery + high dose RT (59.4 Gy)	No significant difference in the 5-years OS between low-dose arm (58%) and high dose arm (59%). No significant difference in the 5-years PFS (47% verss 50%)
NCCTG/RTOG/ECOG (Shaw EG. J Clin Oncol. 2002) [18]	Phase III (*n* = 203)	WHO grade II gliomas	Surgery + low dose RT (50.4 Gy) versus Surgery + high dose RT (64.8 Gy)	No differences in 2- and 5-year OS between low dose (94 and 75%) and high dose arm (85 and 64%) (*p* = 0.48) Patients treated with high doses showed higher rates of severe radionecrosis (5 versus 2.5%)
Adjuvant radiotherapy and chemotherapy				
RTOG 9802 trial (Buckner JC. N Engl J Med. 2016) [21]	Phase III (*n* = 251)	High-risk LGG: Age ≥ 40 years and/or subtotal resection	Postoperative RT (54 Gy) versus Postoperative RT (54 Gy) plus 6 cycles of adjuvant PCV	Post-RT + PCV conferring a survival advantage over RT alone: median OS 13.3 versus 7.8 years (HR: 0.59; 95% CI 0.42–0.83; *p* = 0.003). Median PFS was prolonged in patients who received PCV (10.4 versus 4.0 years): HR: 0.50; 95% CI 0.36–0.68 (*p* < 0.001)
RTOG 0424 (Fisher BJ. J Radiat Oncol Biol Phys. 2015) [23]	Phase II (*n* = 129)	LGG with ≥ 3 risk factors for recurrence (age ≥ 40 years, astrocytoma histology, bihemispheric tumor, tumor diameter > 6 cm, neurologic function status > 1)	Concurrent radiation (54 Gy) with daily temozolomide followed by monthly temozolomide	The 3-year OS rate was 73% (95% CI 65.3–80.8%), significantly higher than the historical control OS rate of 54% (*p* < 0.001). The 5-year OS rate was 57.1% (95% CI 47.7–66.5%), and the median OS has not yet been reached. The 3-year PFS was 59.2% (95% CI 50.7–67.8%) and median PFS was 4.5 years (95% CI 3.5–NA).
Chemotherapy alone with deferred radiotherapy				
EORTC 22033–26033 (Baumert BG. Lancet Oncol. 2016) [24]	Phase III (*n* = 477) stratified by 1p status	LGG with at least one high-risk feature (aged > 40 years, progressive disease, tumour size > 5 cm, tumour crossing the midline, or neurological symptoms)	RT (up to 50.4 Gy versus Dose-dense oral TMZ (75 mg/m^2^ once daily for 21 days, repeated every 28 days, for a maximum of 12 cycles)	There was no significant difference in PFS between TMZ group (39 months) and RT group (46 months): HR: 1.16; 95% CI 0.9–1.5 (*p* = 0.22). Median OS has not been reached. Better PFS in IDH-mutant, non-codeleted patients treated with radiotherapy: 55 versus 36 months. HR 1.86; 95% CI 1.21–2.87 (*p* = 0.0043)
Wahl M, et al. (Neuro Oncol. 2017) [27]	Phase II (*n* = 125)	LGG and gross residual disease	Monthly cycles of TMZ for up to 1 year or until disease progression	The median PFS and OS were 4.2 and 9.7 years, respectively. Patients with 1p/19q codeletion demonstrated a 0% risk of progression during treatment

LGG: low grade gliomas (WHO grade II astrocytoma, oligodendroglioma or mixed oligoastrocytoma)

*RTOG* Radiation Therapy Oncology Group, *EORTC* European Organization for Research and Treatment of Cancer, *MRC* Medical Research Council, *NCCTG* North Central Cancer Treatment Group, *ECOG* Eastern Cooperative Oncology Group study, *OS* overall survival, *PFS* progression-free survival, *TTP* time to progression, *RT* radiotherapy, *PCV* procarbazine, lomustine, and vincristine, *TMZ* Temozolomide

^a^Unfavorable prognostic factors in RTOG 9802 study: tumour size ≥ 4 cm, astrocytoma or oligoastrocytoma histology, and residual disease ≥ 1 cm. RT: radiotherapy

Factors to consider when selecting patients for immediate postoperative therapy include the presence of tumour-related symptoms and risk factors for worse outcome, which comprise age ≥ 40 years, large preoperative tumour size (≥ 4 cm), incomplete resection, astrocytic histology, and absence of 1p/19q-codeletion[11, 12]. It is important to recognize, however, that individual risk factors are relative (including the age cut-off of ≥ 40 years) and exist on a biological continuum. In addition, there is no single agreed-upon definition of low versus high risk, and risk has been variably defined across trials.

Moreover, in the Radiation Therapy Oncology Group (RTOG) 9802 phase II observational study, 111 patients with favorable LGG (< 40 years of age and gross tumor resection, GTR) were observed postoperatively (no adjuvant therapy was given) [13]. The overall survival (OS) rates at 2 and 5 years were 99 and 93%, respectively. However, only 48% of patients remained progression-free at 5 years. Factors associated with a poorer prognosis for progression-free survival (PFS) included large tumour size (≥ 4 cm), astrocytoma or oligoastrocytoma histology, and residual disease ≥ 1 cm by MRI. In those patients with all three unfavorable prognostic factors, the 2- and 5-year PFS rates were 60 and 13%, respectively. If all three favorable prognostic factors were present (< 1 cm residual tumour, tumour diameter < 4 cm, and oligodendroglioma histological type), the 2- and 5-year PFS rates were 100 and 70%, respectively. Patients with a mixture of prognostic factors had an intermediate outcome.

Therefore, a “wait and see” approach following initial surgery may be followed in young patients (≤ 40 years) with a favorable prognosis who have undergone an extensive resection for an IDH-mutant low-grade glioma, especially if molecular studies show the presence of a 1p/19q-codeletion (Level II-B). These tumors have a lower annual growth rate compared to low-grade tumors without 1p/19q-codeletion [14].

Taking into account all these factors, the decision of an immediate or delayed postoperative treatment in patients with LGG could be taken as follows [15]:

- For young patients (≤ 40 years) who undergo complete resection of a tumour with favorable molecular features (IDH mutation with codeletion of 1p19q: oligodendroglioma), we suggest initial observation after surgery. It is expected that these patients will eventually recur and require additional therapy at the time of progression (Level II-B).
- For older patients with residual disease and one or more unfavorable molecular features, we suggest immediate postoperative therapy (Level I).
- For patients who do not fall into any of these categories, the more risk factors they present, the more need immediate postoperative treatment (Level III).

## Radiation therapy

Radiation therapy is part of the standard management for low-grade glioma. There are two areas of controversy, one regarding the optimal time to deliver it, immediately after surgery or delayed after clinical or radiological progression, and another in terms of dose and recommended treatment schedule.

### Timing of radiation

The clinical trial EORTC 22845 [16] randomized 311 patients with low-grade gliomas (WHO grade II or pilocytic astrocytomas with incomplete resection) to receive radiotherapy (54 Gy in 6 weeks) after biopsy or initial resection or not receiving treatment until progression. With a median follow-up of at least 8 years, early radiotherapy was associated with an improvement in progression-free survival (5.3 versus 3.4 years, *p* = 0.0001), with no difference in overall survival (7.4 versus 7.2 years, *p* = 0.872). Seizure control also improved in patients treated with early radiotherapy. Since quality of life was not analyzed, it is not possible to know if time to progression also reflects time to clinical deterioration. The main weak point of this study is the inclusion of both grade II and grade I gliomas, in the absence of a subsequent subgroup analysis, which makes it a Class III study. Karim et al. (level of evidence I study) obtained very similar data in an interim analysis regarding the role of radiotherapy in the results of low grade gliomas [17] (Table 2).

### Dose and schedule

With a level of evidence I, it has been established that low doses of irradiation are equivalent to high doses (45–50.4 versus 59.4–64.8 Gy) in the treatment of low-grade gliomas with postoperative radiotherapy, also reducing treatment toxicity.

The Shaw et al. North American randomized trial compared low irradiation dose (50.4 Gy) versus high (64.8 Gy) for the treatment of 203 patients with supratentorial WHO grade II gliomas. Overall survival was higher but not statistically significant in patients treated with low doses (94 and 75% at 2 and 5 years, versus 85 and 64%, *p* = 0.48). In addition, patients treated with high doses showed higher rates of severe radionecrosis (5% versus 2.5%) [18].

The EORTC 2284 trial [19] randomized 379 patients with low-grade glioma to receive a dose of 59.4 Gy in 33 fractions or 45 Gy in 25 fractions. With a median follow-up of 74 months, no significant differences were found in overall survival or disease-free survival. In contrast, patients treated with high doses showed significantly greater toxicity.

### Side effects

Common acute toxicities include fatigue, hair loss and in some cases increased cerebral oedema. Progressive delayed neurocognitive impairment, when appears, is a consequence both of the disease and its treatment. Besides radiotherapy, the tumor itself, corticoesteroids and antiepileptics could also contribute to this deficit.

The role of radiotherapy in the management of low-grade glioma is clear. With a level of evidence I radiation therapy is recommended in the management of newly diagnosed low-grade glioma in adults to prolong progression-free survival irrespective of extend of resection. Level of evidence II studies recommend that radiation therapy in the management of newly diagnosed low-grade glioma in adults is an equivalent alternative to observation in preserving cognitive function irrespective of extend of resection. With a level of evidence III, radiation therapy improves seizure control in patients with epilepsy and subtotal resection.

## Adjuvant chemotherapy

### PCV (Procarbacine, CCNU, Lomsutine)

There is Level 1 evidence supporting the use of adjuvant chemotherapy following RT. In the RTOG 9802 trial, 251 patients with a supratentorial low-grade glioma were randomly assigned to postoperative RT with or without six cycles of adjuvant PCV (procarbazine, lomustine, and vincristine, see Table 3) chemotherapy[20, 21]. Eligible patients were either age 18–39 years with subtotal resection or biopsy, or age ≥ 40 years with any extent of resection. RT on both trial arms consisted of 54 Gy in 30 fractions, and chemotherapy consisted of six cycles of procarbazine, lomustine, and vincristine (PCV). The patient population included those with diffuse astrocytoma, oligodendroglioma, and mixed astrocytoma/oligodendroglioma in 26, 42, and 32% of cases, respectively.

**Table 3:** PCV (Procarbacine, CCNU, Vincristine) regimen

Drug	Dose
Procarbacine	Days 8–21: 60 mg/m^2^, PO
CCNU (Lomustina)	Día 1: 110 mg/m^2^, VO.
Vincristine	Días 8–29: 1,4 mg/m^2^ IV

Cycle Length: 6 weeks

- The primary endpoint of the trial was overall survival. At the time of the first publication with a median follow-up of 5.9 years, there was a trend toward longer survival in the RT plus chemotherapy group (5-year OS rate 72 versus 63%; HR: 0.72; 95% CI 0.47–1.10). In an update of the data with a median follow-up time of 11.9 years, this difference became significant, with post-RT + PCV conferring a survival advantage over RT alone (median overall survival 13.3 versus 7.8 years; HR: 0.59; 95% CI 0.42–0.83; *p* = 0.003) [21]. Median progression-free survival was also prolonged in patients who received PCV (10.4 versus 4.0 years; HR: 0.50; 95% CI 0.36–0.68; *p* < 0.001).
- The incidence of grade 3 and 4 hematologic toxicity was 8 and 3% in the RT alone arm compared with 51 and 15% in the RT plus PCV arm, respectively. There were no treatment-related deaths and no cases of secondary leukemia [20].
- In an exploratory analysis of overall survival according to individual histologic type, the superiority of radiation therapy plus chemotherapy over radiation therapy alone was seen with all histologic diagnoses, but the magnitude of the effect was greater in patients with oligodendroglioma (*n* = 101; HR: 0.43; 95% CI 0.23–0.82; *p* = 0.009) and oligoastrocytoma (*n* = 77; HR: 0.56; 95% CI 0.32–1.0; *p* = 0.05) than in those with astrocytoma (*n* = 46; HR: 0.73; 95% CI 0.40–1.34) [21]. Nevertheless, a small number of patients in each subgroup preclude definitive conclusions.
- Small numbers and incomplete data have limited post hoc molecular analysis of the RTOG 9802 trial. IDH1 R132H mutation was present in 61% (35/57) of patients treated with RT + PCV and in 64% (36/56) of those treated with RT. Patients with tumoral IDH1 R132H mutations had significantly longer overall survival than did those without the mutation, regardless of treatment (*p* = 0.02). The median overall survival was 13.1 years (95% CI 10.1 to not-reached) among patients with the mutation versus 5.1 years (95% CI 1.9–11.5) among those without the mutation. Among patients with tumoral IDH1 R132H mutations, those who received radiation therapy plus chemotherapy had longer overall survival than did those who received radiation therapy alone (HR: 0.42; 95% CI 0.20–0.86; *p* = 0.02). The number of events among patients without the IDH1 R132H mutation was too small to determine the association of treatment effect in this subgroup.

Moreover, analogous randomized trials in patients with anaplastic gliomas (i.e., RT with or without adjuvant PCV) found that the survival advantage of PCV was largely driven by patients with oligodendroglial tumors with 1p/19q-codeletion, and possibly others (e.g., 1p/19q-non-codeleted tumors with MGMT methylation or an IDH1 mutation) [22].

### Temozolomide

The best available data on the safety and efficacy of chemo-radiation with temozolomide in patients with high-risk low-grade glioma comes from a phase II multicenter RTOG 0424 study (Level 3 of evidence), in which 129 patients with low-grade glioma with three or more risk factors for recurrence (age ≥ 40 years, astrocytoma histology, bihemispheric tumor, preoperative tumor diameter > 6 cm, preoperative neurologic function status > 1) were treated with concurrent radiation (54 Gy in 30 fractions) with daily temozolomide followed by monthly temozolomide [23]. At a median follow-up time of 4.1 years, 3-year overall survival (OS) rate was 73% (95% CI 65.3–80.8%), significantly higher than the historical control OS rate of 54% (*p* < 0.001). The 5-year OS rate was 57.1% (95% CI 47.7–66.5%), and the median survival time (MST) has not yet been reached. The 3-year PFS was 59.2% (95% CI 50.7–67.8%), and median PFS was 4.5 years (95% CI 3.5–NA) (Level III–C). The majority of adverse events (AEs) were hematologic and grade 3–4 AEs occurred in 43 and 10% of patients, respectively. There were no reported cases of radiation necrosis or second malignancy within the available follow-up time. One patient experienced a grade-5 infection (herpes encephalitis) possibly related to TMZ or steroids. Neurocognitive and quality of life outcomes were not presented.

Whether temozolomide should replace PCV is not clear. Among patients with anaplastic tumors, it has already done so, as it is undoubtedly less toxic and easier both to prescribe and receive. An interim analysis of the CATNON study of patients with 1p/19q-non-codeleted anaplastic glioma found that 12 cycles of adjuvant temozolomide improved survival over radiation alone (HR 0.67; 95% CI 0.51–0.88; 5-year OS 56 versus 44%) [24].

However, temozolomide may not be as efficacious as PCV in low-grade gliomas. For example, median survival in RTOG 0424 has not been reached, but early results demonstrated median PFS of 4.5 years and a 3-year PFS rate of 59%. These results with temozolomide appear inferior to those with PCV from RTOG 9802 in which median PFS was 10.4 years and the 3-year PFS rate was 75–80%, although cross-trial comparisons are fraught with difficulty because of differences in entry criteria and study populations.

### Chemotherapy alone with deferred radiotherapy

One option for patients with favorable clinical and molecular factors is to start with chemotherapy and reserve a decision about RT until response is determined. Part of the rationale for such an approach is to avoid potential short- and long-term toxicities of radiation for as long as possible.

A NCCTG/Mayo Clinic trial treated 28 patients with low-grade Oligodendroglioma or Oligoastrocytoma who had residual disease postoperatively with an intensified regimen of PCV, to determine the response rate and toxicity of PCV administered before radiation therapy [25]. Radiation therapy (59.4 or 54.0 Gy) began within 10 weeks of completing chemotherapy or immediately if there was evidence of tumour progression on PCV.

The response rate observed by clinicians was 29% (95% CI 13–49%). Blinded independent single neuroradiologist verified tumour regression in 13 of 25 eligible patients (52%; 95% CI 31–72%).

Myelosuppression was the predominant toxicity: grade 3–4 leukopenia occurred in 75% of patients and grade-3 thrombocytopenia was recorded for 64%. Neurologic toxicity consisted of lethargy and sensory changes related to peripheral neuropathy, was predominantly mild to moderate.

At the time of this analysis, 3 of the 28 patients (11%) had died, and follow-up in the remaining 25 living patients ranged from 3.24 to 7.38 years, with a median of 4.83 years. Progressions had been recorded for seven (25%) of the participants. Kaplan–Meier estimates of the percentage alive at 1, 2, and 5 years were 100, 96, and 89%, respectively. Kaplan–Meier estimates of the percentage recurrence free at 1, 2, and 5 years were 91%, 62%, and undefined, respectively.

Loss of 1p and 19q seems limited to patients with pure oligodendroglioma (*p* = 0.009) and were not associated with response to PCV (possibly because of the small sample size).

The most robust data on single-modality treatment of low-grade glioma come from the EORTC 22,033–26,033 trial, that compared standard radiotherapy versus primary temozolomide chemotherapy, in a phase III trial that randomized 477 patients who had a low-grade (WHO grade II) glioma (astrocytoma, oligoastrocytoma, or oligodendroglioma) with at least one high-risk feature (aged > 40 years, progressive disease, tumour size > 5 cm, tumour crossing the midline, or neurological symptoms) [26]. Eligible patients were stratified by 1p status and then were randomly assigned (1:1) to receive either conformal radiotherapy (up to 50.4 Gy; 28 doses of 1.8 Gy once daily, 5 days per week for up to 6.5 weeks) or dose-dense oral temozolomide (75 mg/m^2^ once daily for 21 days, repeated every 28 days, for a maximum of 12 cycles).

At a median follow-up of 48 months (IQR 31–56), there was no significant difference in progression-free survival between two treatments: median PFS survival was 39 months (95% CI 35–44) in the temozolomide group and 46 months (95% CI 40–56) in the radiotherapy group (HR: 1,16; 95% CI 0.9–1.5, *p* = 0.22). Median overall survival has not been reached.

Exploratory analyses in 318 molecularly defined patients confirmed the significantly different prognosis for progression-free survival in the three recently defined molecular low-grade glioma subgroups: IDH mutation with or without 1p/19q codeletion, or IDH-wildtype (*p* = 0.013).

- In the subgroup of patients with IDH-mutant, 1p/19q-non-codeleted tumors, progression-free survival was longer in patients randomized to receive radiation (55 versus 36 months; HR 1.86; 95% CI 1.21–2.87; *p* = 0.0043) (Level II–B).
- In contrast, progression-free survival was similar for radiation versus temozolomide in patients with 1p/19q-codeleted tumors (62 versus 55 months) and in patients with IDH-wildtype tumors (19 versus 24 months). Level II-B).
- Further data maturation is needed for overall survival analyses and evaluation of the full predictive effects of different molecular subtypes for future individualized treatment choices.

In a phase II study of adjuvant temozolomide [13, 27], 0 patients with low-grade gliomas and gross residual disease after surgical resection received monthly cycles of temozolomide for up to 1 year or until disease progression. For patients with available tissue, molecular subtype was assessed based upon 1p/19q codeletion and IDH-1 R132H mutation status. With a median follow-up of 7.5 years, the median PFS and OS were 4.2 and 9.7 years, respectively. Patients with 1p/19q codeletion demonstrated a 0% risk of progression during treatment. Patients with 1p/19q codeletion are potential candidates for the omission of adjuvant radiotherapy, but further work is needed to directly compare chemotherapy with combined modality therapy.

It is important to note, however, that even a median progression-free survival of 4–5 years in 1p/19q-codeleted tumors with adjuvant temozolomide [24, 27], is far inferior to the approximately 10-year progression-free survival achieved by RT plus PCV in the RTOG 9802 study [21]. In addition, whether RT with delayed chemotherapy delivered at the time of relapse can garner the same survival advantage as adjunctive chemotherapy delivered with RT is not yet known, and long-term follow-up data on overall survival as well as other endpoints, such as cognition and quality of life, are needed.

Therefore, postoperative chemotherapy with delayed radiation can be considered in patients with 1p/19q-codeleted oligodendrogliomas for whom postoperative therapy is indicated, although long-term outcome data are more limited for this approach compared with early RT with or without chemotherapy (level II).

### Treatment IDH-wildtype low-grade gliomas

Low-grade gliomas that lack a mutation in IDH make up a relatively small proportion of all low-grade gliomas and carry a significantly worse prognosis compared with IDH-mutant tumors [28].

There are no randomized trials to guide treatment in patients with IDH-wildtype low-grade gliomas, and these tumors are underrepresented in historical studies of low-grade glioma, including the RTOG 9802 trial reviewed above.

Some of these tumors bear molecular similarity to glioblastoma (e.g., TERT mutations, loss of heterozygosity of chromosome 10). In such cases, we propose to treat patients with immediate postoperative therapy, regardless of extent of resection or other prognostic factors, with radiation and chemotherapy. Whether to treat IDH-wildtype with RT + PCV or with concurrent chemorradiotherapy with TMZ followed by adjuvant TMZ is an open question that requires further studies. The rational to use the same regimen as in glioblastoma, the Stupp regimen [29], is the fact that IDH-wt astrocytoma has the same biology and natural history is very similar to primary GBM (Level III).

## Recommendations for follow-up

There are no formal clinical trials that define the optimal frequency for follow-up after treatment.

Follow-up with FLAIR or T2-weighted MRI is the standard of care. These MRI sequences are widely available and can be used routinely in clinical protocols. Routine follow-up should also include T1-weighted images before and after intravenous contrast administration to detect malignant transformation.

In a phase III trial on dose response of LGG to RT, a quality of life (QoL) questionnaire was included. Analysis of responses suggested that patients who received high-dose RT had lower levels of daily functioning and more negative symptoms after RT [30]. Neuropsychological tests, at diagnosis and during the follow-up, can be useful, being selected according to the needs of the clinical setting.

Follow-up recommendation would be clinical evaluation with particular attention to neurological function, seizures and corticosteroid use. Patients should be tapered off steroid use as early as possible. Venous thrombotic events occur frequently in patients with residual or recurrent tumours. Laboratory tests are not indicated unless the patient is receiving chemotherapy, corticosteroids or anti-epileptic drugs. MRI and Mini–Mental State Examination (MMSE) after the completion of therapy, and then every 3–4 months for 1 year, every 6 months for 2 years, and every year thereafter until tumor progression is recommended outside clinical trials (Level V), unless more frequent monitoring is clinically indicated.

## Summary of recommendations

**Fig. 3:**
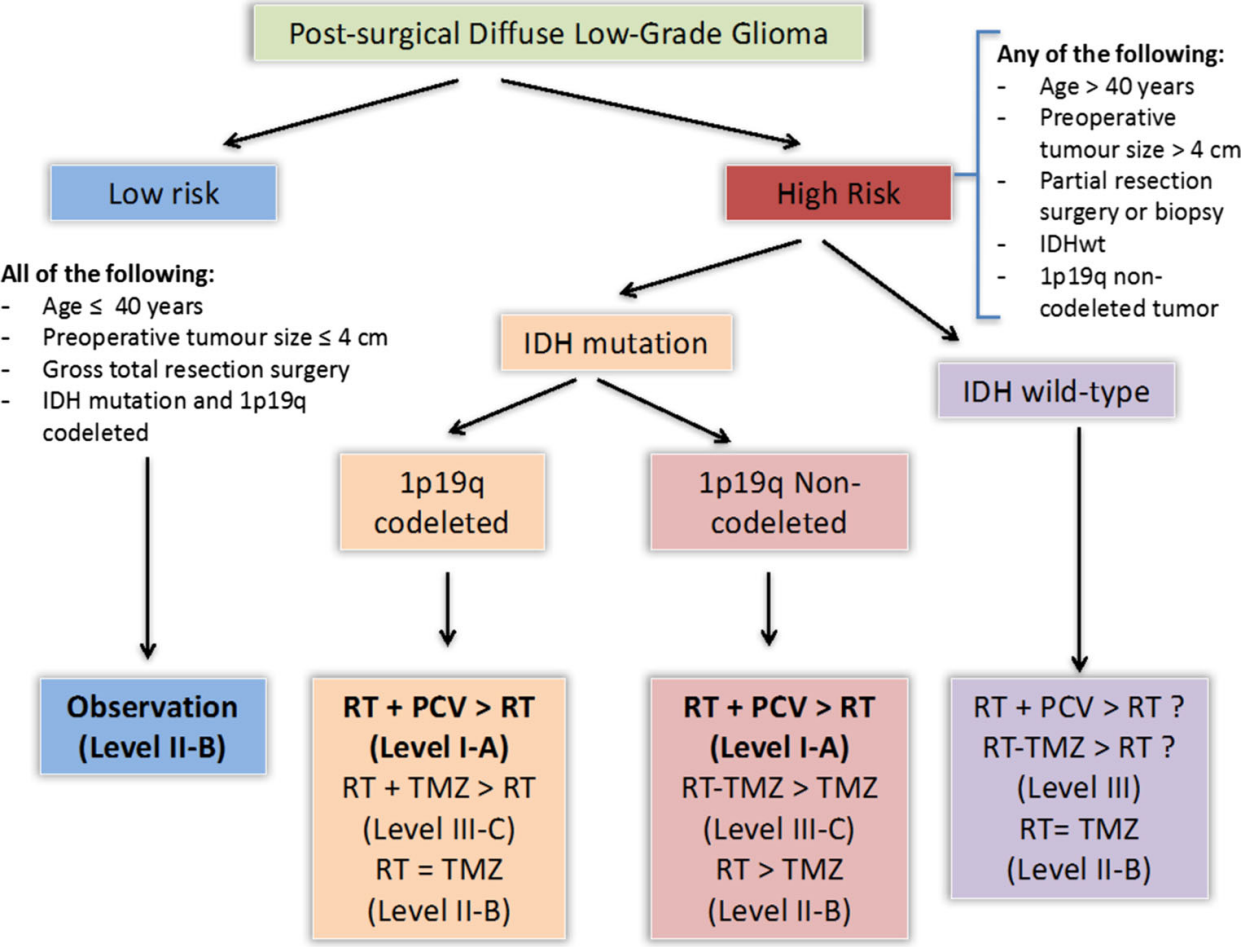
Therapeutic algorithm for difuse low-grade gliomas

Table 4 summarizes the global recommendations of this guideline. Figure 3 shows the therapeutic algorithm of LGG.

**Table 4:** SEOM guideline recommendations for low grade glioma

Diagnosis and classification -Histological evaluation is based on the 2016 WHO Classification that integrates molecular markers in the routine histological diagnosis: IDH mutation and 1p/19q codeletion
-MRI is the modality of choice for characterizing brain tumors. Level of evidence: III. Grade of recommendation: A
Surgery in low grade glioma -Tumor removal with greater extent of resection when feasible is recommended. Level of evidence II. Grade of recommendation: A -Biopsy is indicated when diagnosis is needed in deep lesions (including brainstem), diffuse and/or multicentric tumor or any other contraindication for open resection. Biopsy can be stereotactic or open -In incidental LGG surgical resection is superior when compared with observation to improve overall survival. Level of evidence III. Grade of recommendation: XXX Timing of complementary treatment after surgery -For young patients (≤ 40 year-old) who undergo complete resection of a tumour with favorable molecular features (IDH mutation with codeletion of 1p19q: oligodendroglioma), we suggest initial observation after surgery. It is expected that these patients will eventually recur and require additional therapy at the time of progression. Level of evidence: III. Grade of recommendation: B -For patients older than 40 year-old with residual disease and one or more unfavorable molecular features, we recommend immediate postoperative therapy. Level of evidence I. Grade of recommendation: A For patients who do not fall into any of these categories, the more risk factors they present, the more need immediate postoperative treatment. Risks factors associated with a poorer prognosis for PFS: large tumour size (≥ 4 cm), astrocytoma or oligoastrocytoma histology, and residual disease ≥ 1 cm by MRI. Level of evidence: III. Grade of Recommendation: B Complementary treatment -Low doses of irradiation are equivalent to high doses (45–50.4 versus 59.4–64.8 Gy) in the treatment of low-grade gliomas with postoperative radiotherapy, also reducing treatment toxicity. Level of Evidence: I. Grade of Recommendation: A -The use of six cycles of adjuvant chemotherapy following RT with PCV (Procarbazine, Lomustine and Vincristine) improves overall survival. Level of Evidence: I. Grade of Recommendation: A - Whether temozolomide should replace PCV is not clear. However, temozolomide may not be as efficacious as PCV in low grade gliomas. Grade of Recommendation: C Follow-up -Follow-up with FLAIR or T2-weighted MRI is the standard of care. These MRI sequences are widely available and can be used routinely in clinical protocols. Routine follow-up should also include T1-weighted images before and after intravenous contrast administration to detect malignant transformation

## Conclusions

1. The revised WHO classification of 2016 integrates molecular markers in the routine histological diagnosis of CNS tumors.
2. The main molecular factors for subdividing LGG are IDH mutations, 1p19q codeletions, ATXR and TERT mutations.
3. LGG may be subdivided into three groups: (1) Grade 2 diffuse astrocytoma IDH wt, (2) Grade 2 diffuse Astrocytoma IDH mut without 1p19q codeletion, (3) Grade 2 Oligodendroglioma that requires the following molecular features: IDH mut + 1p19q codeletion.
4. MRI is the modality of choice for characterizing LGG but advanced MRI techniques, such as Diffusion weigthed imaging (DWI), MR Spectroscopy and Perfusion MRI, complements the anatomic information obtained from conventional MRI.
5. Surgical resection is considered the first step to be done when dealing with LGG. Currently is assumed that surgery should aim for the greater extent of resection.
6. As the management of LGG is complex, It is recommended that all cases of low-grade glioma be discussed in a multidisciplinary committee before selecting a therapeutic option.
7. Figure 3 summarizes the recommendation for the multidisciplinary postsurgical management of LGG.
